# Expression profiling of small intestinal neuroendocrine tumors identified pathways and gene networks linked to tumorigenesis and metastasis

**DOI:** 10.1042/BSR20193860

**Published:** 2020-06-16

**Authors:** Qiang Wang, Chaoran Yu

**Affiliations:** 1 Fudan University Shanghai Cancer Center, Fudan University, Shanghai 200025, P.R. China; 2 Department of Oncology, Shanghai Medical College, Fudan University, Shanghai 200025, P.R. China

**Keywords:** Differentially expressed genes, Gene ontology, Gene set enrichment analysis, KEGG pathway, Protein-protein interaction network, Small intestinal neuroendocrine tumors

## Abstract

Small intestinal neuroendocrine tumors (SI-NETs) remain the most common subset in gastrointestinal neuroendocrine tumors and featured by aggressiveness. However, the molecular feature of SI-NETs remains largely unclear with key genes and pathways yet to be identified. The gene expression profile GSE65286 was retrieved for analysis. Artificial neural networks (ANNs) were constructed for the hub genes network models. A total of 613 differentially expressed genes (DEGs) were identified between normal (N) and primary tumor (T) groups, whereas 61 DEGs were identified between T and liver metastases (LM) groups. The top Kyoto Encyclopedia of Genes and Genomes (KEGG) pathways for the DEGs of N versus T were fat digestion and absorption pathway. For T versus LM the top KEGG pathways were complement and coagulation. In gene set enrichment analysis (GSEA), five gene sets, including Notch signaling, inflammatory response, coagulation, KRAS signaling, and allograft rejection were significantly enriched in the T group. The hub genes in the DEGs of T versus LM included albumin, fibrinogen gamma chain, alpha 2-HS glycoprotein, transferrin and GC, vitamin D binding protein. A distinct correlational alteration of hub genes was observed between T and LM groups. In ANN analysis, ALB and TF were the top predictors of metastasis. Moreover, the expression of ALB≤ showed the highest support to T whereas ALB>15.97 supports LM. TF≤7.54 showed the highest negative correlation to the T. This bioinformatics analysis provided insights on potential key pathways and genes networks involved in SI-NETs and established an ANN-based hub gene model for metastatic prediction.

## Introduction

Neuroendocrine tumors (NETs) were initially described by Oberndofer using karzinoid (carcinoid) in 1907 and mostly arise from the neuroendocrine cells with the ability to generate hormonal-related peptides [1,2]. Small intestinal neuroendocrine tumors (SI-NETs), the most common subset in gastrointestinal neuroendocrine tumors, are featured by aggressiveness and therapy-resistance [1,3]. During the last three decades, the incidence of SI-NETs has annually increased by 3.8% [4]. In fact, the age-adjusted incidence of NETs (over 30 years old) in digestive system has dramatically increased by 720%, with 225% increasing in ileal part and 460% in small intestinal [4]. However, the five-year survival rate surprisingly remains unchanged [4].

Of note, SI-NET patients commonly are diagnosed as advanced stage at initial presentation due to indolent unnoticeable clinical course [1]. Numerous terms have been associated with the prognosis of SI-NET, including age, carcinoid heart diseases, lymph node metastases, liver tumor load, peritoneal carcinomatosis, and tumor cell proliferation (WHO grade) [2,5,6]. Moreover, the somatostatin analogs treatment has significantly improved the tumor progression in well-differentiated metastatic midgut NETs [7].

Despite the fact that therapeutic progressions have been made, the overall clinical benefits of SI-NETs remain far from satisfactory. Reasonably, simplified therapeutic management of SI-NETs does not fully capture the full biological picture. In fact, molecular characterization of SI-NETs would contribute to the development of novel therapeutic strategies. Interestingly, frameshift mutation of CDKN1B (encoding p27) was found in 7.8% SI-NETs patients, indicating the role of cell cycle dysregulation involved [8]. In addition, nine miRNAs were found differentially expressed during tumor progression (miR-96, -182, -183, 196a, -200a, -31, -129-5p, -133a, and -215) [9]. Moreover, a subset of SI-NETs clustered by transcriptome files was characterized by longer survival and higher expression of SSTR2, whereas shorter survival was associated with higher grade or gain of chromosome 14 [5]. However, the molecular picture of SI-NETs remains largely unclear.

In the present study, the gene expression profile GSE65286, deposited by Andersson co-workers, was analyzed using bioinformatics strategy [5], followed by functional enrichment analysis of differentially expressed genes (DEGs) and the identification of key genes and pathways.

## Materials and methods

### Gene expression profile

The gene expression profile GSE65286, containing 10 primary tumors (T), 10 normal small intestine mucosa (N), 2 lymph nodes metastases (LN), 21 tumor with liver metastases samples (LM), was retrieved from the Gene Expression Omnibus (GEO) (http://www.ncbi.nlm.nih.gov/geo/) [5,10]. All the RNA was retrieved from the fresh-frozen samples using the miRNeasy Mini Kit (Qiagen) and synthesized and labeled following the One-Color Microarray-Based Gene Expression Analysis protocol (v5.7). Next, the Whole Human Genome Microarrays (GPL4133, G4112F, ID: 014850, Agilent Technologies) were used for hybridization. The scanning process was performed using Agilent Microarray Scanner G2565BA (Agilent Technologies) and the results were processed by Feature extraction version 10.7.1.1 (Agilent Technologies) with normalization [5].

### Identification of DEGs and functional enrichment

Identification of DEGs was performed by the GEO2R analytical tool [11]. The cut-off values of DEGs were defined by the adj. *P*-value<0.05 (Benjamini and Hochberg’s False discovery rate, FDR,) and |log2 fold change (logFC)|>2. The Kyoto Encyclopedia of Genes and Genomes (KEGG) and gene ontologies (GO), including biological processes (BP), cellular components (CC) and molecular functions (MF), were annotated by R package ClusterProfiler and the Database for Annotation, Visualization and Integrated Discovery (DAVID) [12–14].

### Gene set enrichment analysis

The gene set enrichment analysis (GSEA) was performed using the Broad Institute GSEA software (http://software.broadinstitute.org/gsea/index.jsp) [15] with the annotation file “hallmark gene sets” and default cutoff values (*P*-value<0.05).

### Construction of protein–protein interaction networks

The protein–protein interaction (PPI) networks were established by the DEGs interactions generated by the Search Tool for the Retrieval of Interacting Genes/Proteins (STRING http://www.string-db.org/). The minimum required interaction score was set as medium confidence (0.400). Active interaction sources included textmining, experiments, databases, co-expression, neighborhood, gene fusion and co-occurrence. The results were visualized via the Cytoscape software (version 3.6.0) [16,17]. Hub genes were defined by the top 10 genes with highest degree values. The Molecular Complex Detection (MCODE) was used to subset the PPI networks [18].

### Artificial neural networks for hub gene network models

Artificial neural networks (ANNs) are characterized by non-linear mathematical models with highly parameterized input for the description of complex systems [19–21]. Unlike conventional linear regression models, ANNs are featured by “black boxes” powerful algorithms [21]. In fact, ANNs generate challenging networks that could approximate outcomes with minimal errors, however, with abstract interpretations [19–21]. In the present study, ANNs were used to predict the metastasis status of SI-NETs with the input (I1–I5) of previously identified hub genes (ALB, FGG, AHSG, TF, and GC) and the output was the metastasis status (O1, 0 = non metastasis, 1 = metastasis). The hidden process in the complex mathematical models was illustrated by H1–H5 (the number of hidden units is selected based on the best calculated accuracy). All the hidden nodes further led to the output (O1). All the bias factors were represented by bias nodes (B1, B2) similar to the intercept term in a linear model. All the relative importance of hub genes and the metastasis were identified using the Garson’s algorithm in the NeuralNetTool package (version 1.5.1) in R software [22].

## Results

### Identification of DEGs

**Figure 1:**
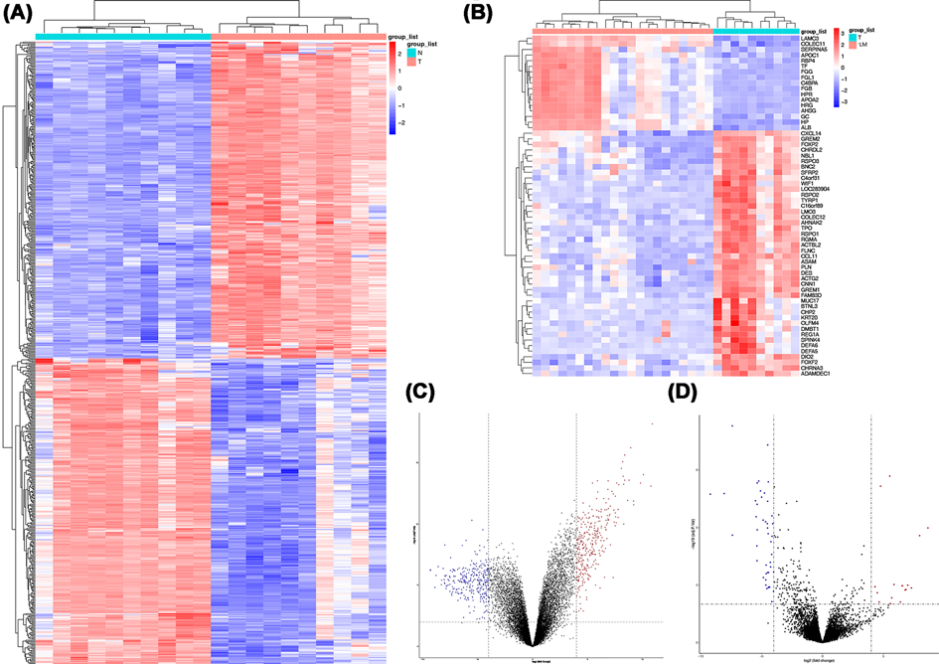
Identification of DEGs in primary tumors (T) versus normal (N) group, as well as T versus liver metastases (LM) group (**A**) The heat map of DEGs in T versus N group; (**B**) the heat map of DEGs in T versus LM group; (**C**) the volcano plot of DEGs in T versus N group; (**D**) the volcano plot of DEGs in T versus LM group.

Given GSE65286 only contained 2 LN samples, the comparison only performed in N (*n* = 10) versus T (*n* = 10) and T (*n* = 10) versus LM (*n* = 21). A total of 613 DEGs were identified between N and T, with 301 down-regulated and 312 up-regulated. Sixty-one DEGs were identified between T and LM, with 44 down-regulated and 17 up-regulated (Figure 1A–D).

### GO and KEGG enrichments

**Figure 2:**
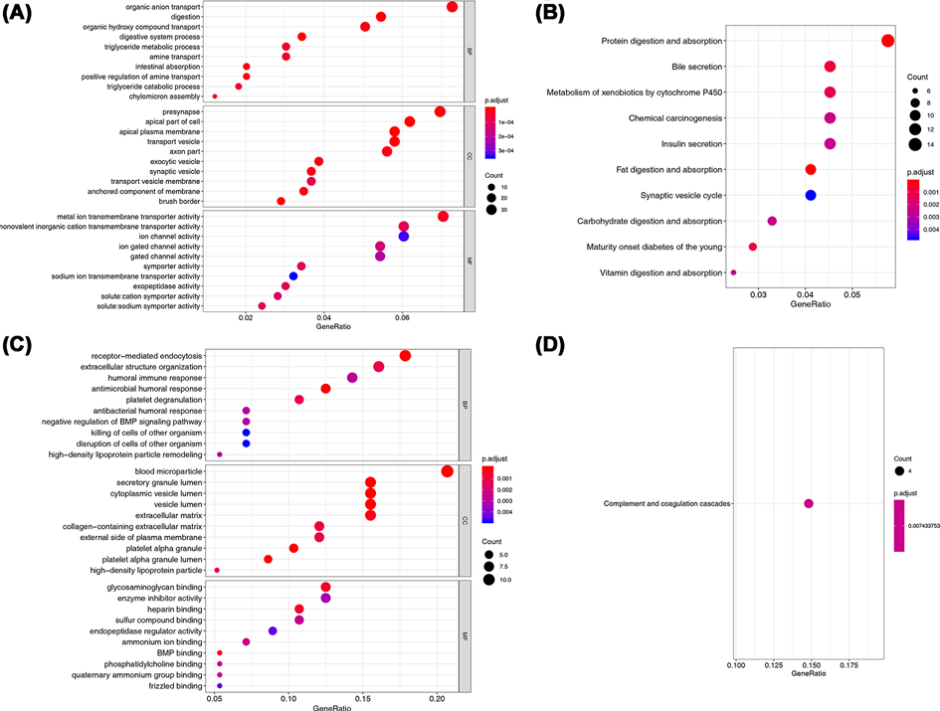
GO and KEGG results of DEGs in T versus N and T versus LM groups (**A**) GO enrichments of DEGs in T versus N group; (**B**) KEGG pathways of DEGs in T versus N group; (**C**) GO enrichments of DEGs in T versus LM group; (**D**) KEGG pathways of DEGs in T versus LM group.

For DEGs between N and T, digestion (*P*-value = 8.09E−13), apical plasma membrane (*P*-value = 3.11E−07), and metal ion transmembrane transporter activity (*P*-value = 3.00E−05) were the top significantly enriched terms in BP, CC, and MF, respectively (Figure 2A). In terms of pathway, fat digestion and absorption, protein digestion and absorption as well as maturity onset diabetes of the young were the top enriched pathways in KEGG (Figure 2B). For DEGs between T and LM, antimicrobial humoral response (*P*-value = 5.41E−05), blood microparticle (*P*-value = 1.75E−12), and BMP binding (*P*-value = 0.000501) were the top significantly enriched terms in BP, CC, and MF, respectively (Figure 2C). However, the complement and coagulation cascades was the only significantly enriched KEGG pathway between T and LM (Figure 2D).

### GSEA

**Figure 3:**
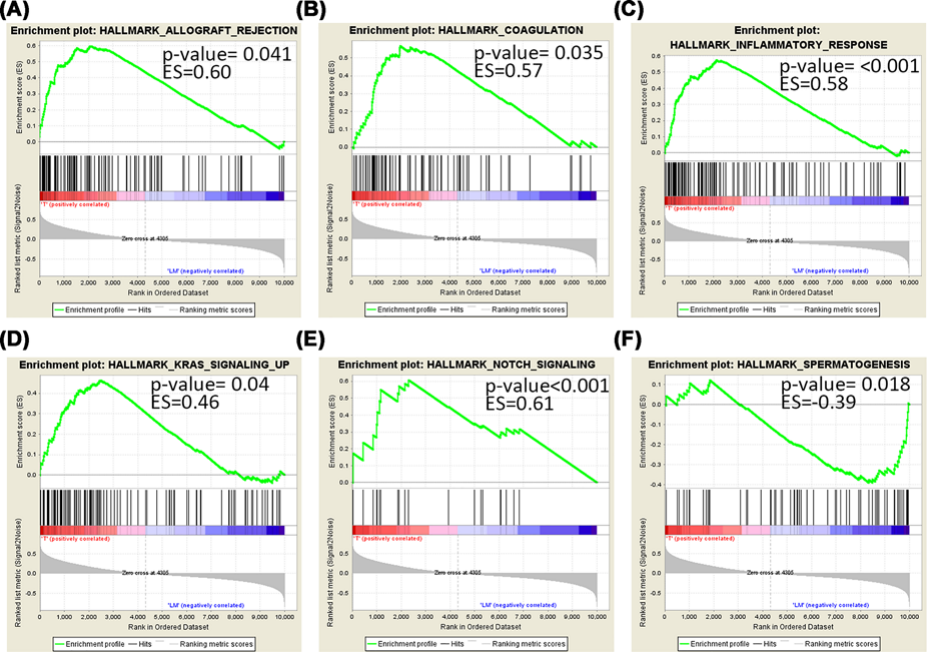
GSEA of primary tumors (T) versus liver metastases (LM) groups (**A**–**E**) Significantly enriched pathways in T group; (**F**) significantly enriched pathway in LM group.

In GSEA, there were five gene sets significantly enriched in the tumor group and one gene set in the liver metastasis group, whereas no significantly enriched gene set between normal and tumor groups. In the liver metastasis group, only spermatogenesis was significantly enriched (*P*-value = 0.018, enrichment score (ES) = −0.39). In the tumor group, the five gene sets included Notch signaling (*P*-value<0.001, ES = 0.61), inflammatory response (*P*-value<0.001, ES = 0.58), coagulation (*P*-value = 0.035, ES = 0.57), KRAS signaling (*P*-value = 0.04, ES = 0.46), and allograft rejection (*P*-value = 0.041, ES = 60) (Figure 3).

### PPI networks construction

**Figure 4:**
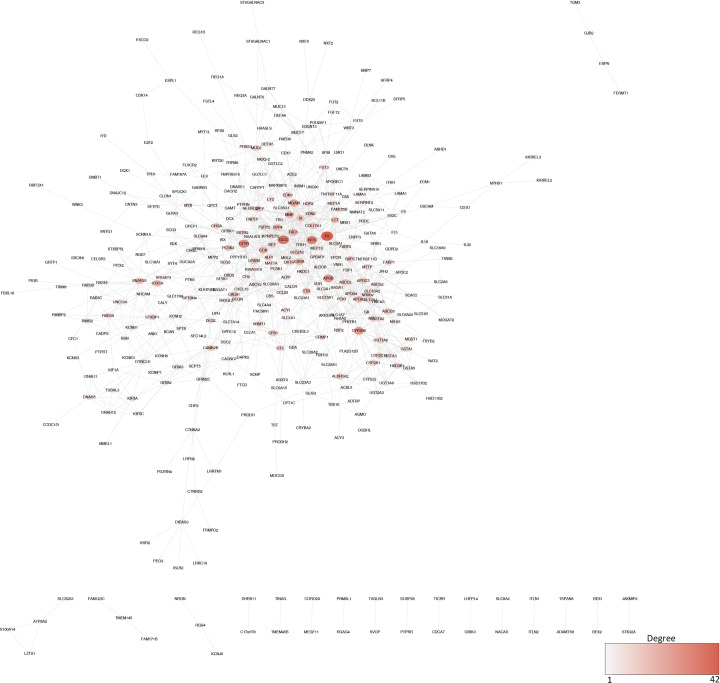
PPI networks of DEGs in T versus N group The degree of each node was reflected by the color and the size. The higher the degree, the darker and bigger the node is.

**Figure 5:**
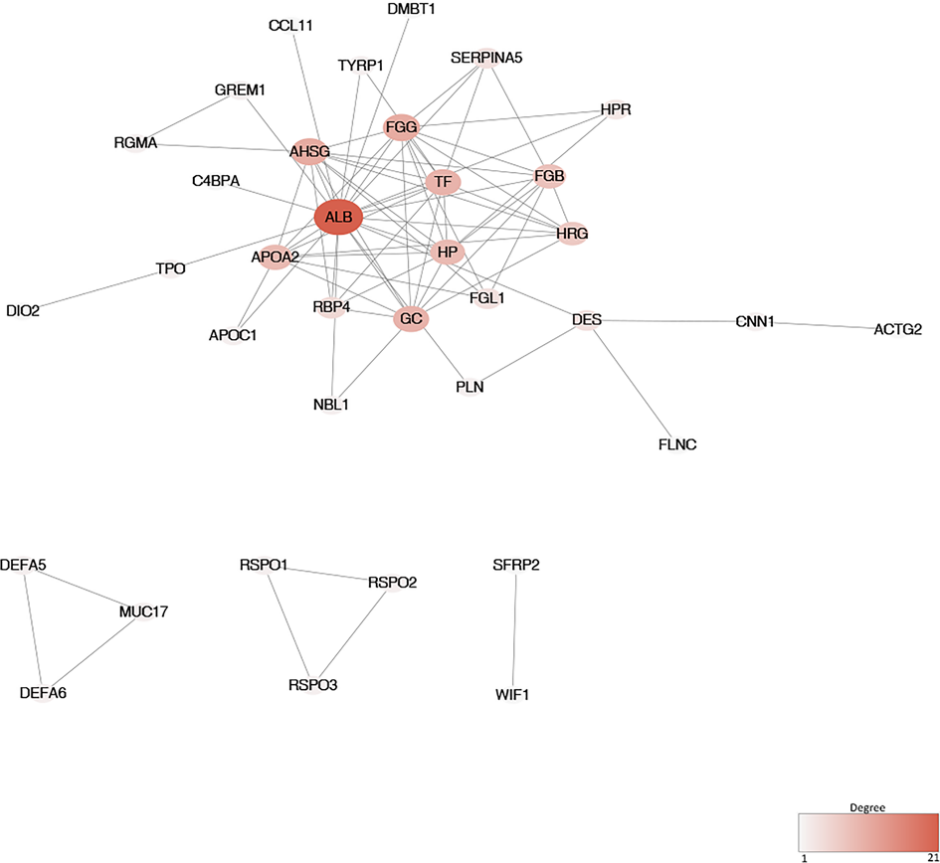
PPI networks of DEGs in T versus LM group The degree of each node was reflected by the color and the size.

The PPI networks in T versus N group (PPI-TvsN) contained 395 nodes with 1046 edges whereas the PPI networks constructed in T versus LM (PPI-TvsLM) contained 36 nodes with 76 edges (Figures 4 and 5). Top three subsets were identified by MCODE in Both PPI networks with significantly enriched pathways (Table 1). The hub genes in the PPI-TvsN included coagulation factor II, thrombin (F2), glucagon (GCG), neurotensin (NTS), cystic fibrosis transmembrane conductance regulator (CFTR) and apolipoprotein B (APOB) (Table 2). The hub genes in the PPI-TvsLM included albumin (ALB), fibrinogen gamma chain (FGG), alpha 2-HS glycoprotein (AHSG), transferrin (TF), and GC, vitamin D binding protein (GC) (Table 2).

**Table 1:** The MCODE with significantly enriched pathways

	Cluster ID	Nodes	Edges	Node IDs	KEGG pathways	*P*-value
PPI TvsN	1	11	55	P2RY2, OXTR, EDN2, LPAR5, NTS, TAC1, CCK, GCG, EDN3, CASR, F2	hsa04080: neuroactive ligand–receptor interaction	0.009186
	2	28	84	APOA4, MGST1, XPNPEP2, GSTA1, APOC2, ABCG5, CYP2S1, ACE2, MME, MEP1A, MEP1B, ADCY2, SHBG, DPP4, GSTA5, GSTA2, FABP2, CYP2B6, MTTP, CYP2C18, APOC3, CXCL13, CCL25, OPRK1, APOB, APOA1, SSTR2, GSTA3	hsa00980: metabolism of xenobiotics by cytochrome P450	1.90E−07
					hsa04975: fat digestion and absorption	2.20E−07
					hsa00982: drug metabolism – cytochrome P450	3.71E−06
					hsa05204: chemical carcinogenesis	8.30E−06
					hsa04974: protein digestion and absorption	1.33E−05
					hsa00480: glutathione metabolism	3.02E–05
					hsa04977: vitamin digestion and absorption	0.002802
					hsa00830: retinol metabolism	0.022278
					hsa03320: PPAR signaling pathway	0.024271
	3	5	9	MUC17, GALNT6, MUC13, MUC2, GALNT7	hsa00512: mucin type O-glycan biosynthesis	0.008993
PPI T vsLM	1	9	32	APOA2, FGG, HP, AHSG, ALB, HRG, TF, GC, RBP4	–	–
	2	3	3	MUC17, DEFA5, DEFA6	–	–
	3	3	3	RSPO1, RSPO2, RSPO3	–	–

The MCODE was an embedded algorithm in the Cytoscape software (version 3.6.0); T: tumor; N: normal; LM: liver metastases. Node IDs: represent each gene symbol.

**Table 2:** Hub genes in PPI networks of tumor versus normal groups and tumor versus liver metastases groups

	Gene symbol	Gene name	Degree
PPI T versus N	F2	Coagulation factor II, thrombin	42
	GCG	Glucagon	36
	NTS	Neurotensin	32
	CFTR	Cystic fibrosis transmembrane conductance regulator	30
	APOB	Apolipoprotein B	27
PPI T versus LM	ALB	Albumin	21
	FGG	Fibrinogen gamma chain	11
	AHSG	Alpha 2-HS glycoprotein	11
	TF	Transferrin	10
	GC	GC, vitamin D binding protein	10

T: tumor; N: normal; LM: liver metastases; degree: represents the connection between each two genes.

### Correlations of hub genes

**Figure 6:**
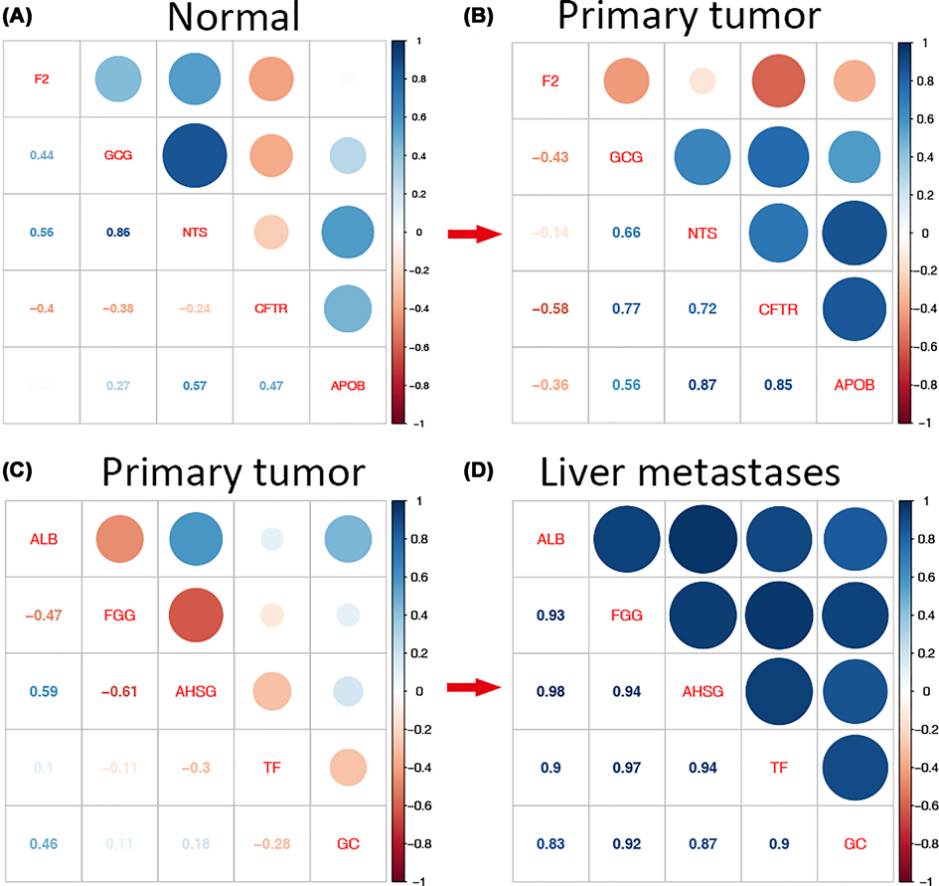
Hub gene correlations (**A,B**) The pairwise correlations of hub genes identified by DEGs in T versus N groups; (**C,D**) the pairwise correlations of hub genes identified by DEGs in T versus LM groups.

Next, the pairwise correlation of hub genes in both PPI networks was analyzed respectively in each phenotype (Figure 6A–D). Remarkably, the highest correlation observed between GCG and NTS (cor = 0.86) in N group was altered in T group, with the correlation between NTS and APOB as the highest one (cor = 0.87). Meanwhile, the correlation of CFTR and APOB, as well as CFTR and GCG, CFTR and NTS were increased distinctly (Figure 6A,B). Noteworthy, the correlation of hub genes between T and LM displayed a dramatic positive change (Figure 6C,D). In fact, given the distinct correlational alterations of hub genes between T and LM groups, these hub genes were chosen for further ANN analysis.

### ANN for hub genes models

**Figure 7:**
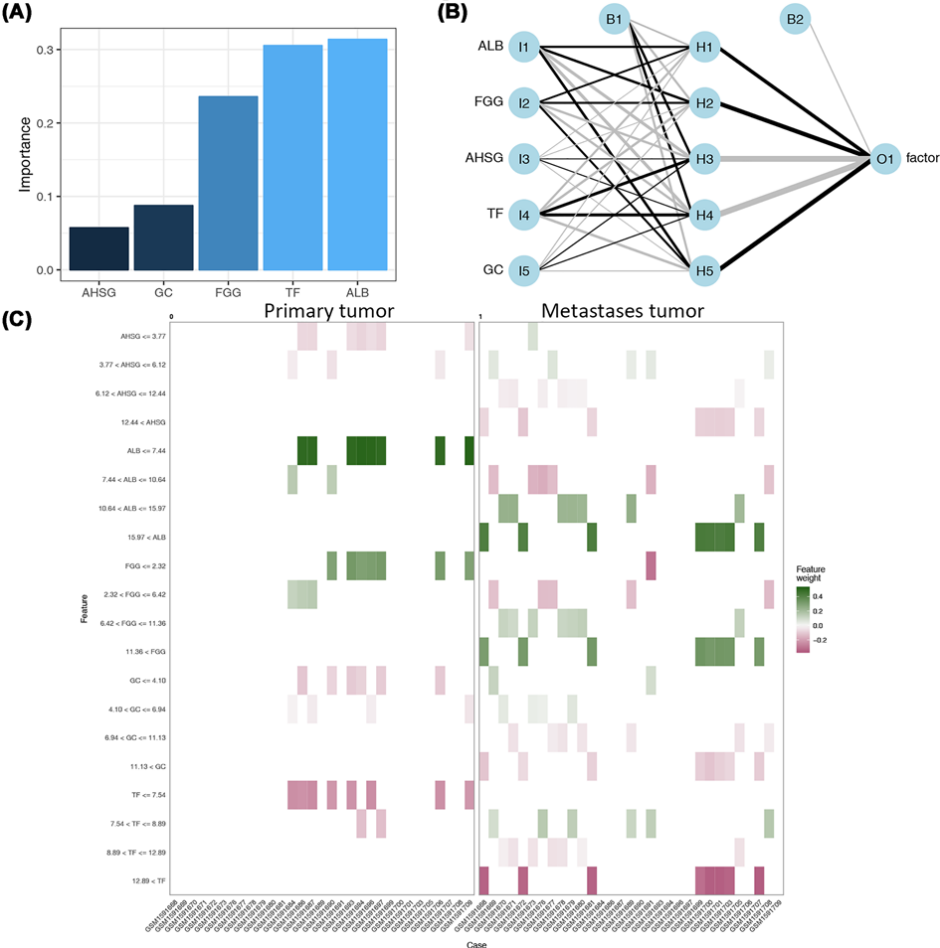
ANN models for hub genes in T versus LM groups (**A**) Relative importance of hub genes to metastasis status; (**B**) ANN interpretation diagram of hub genes to metastasis outcome; positive correlation was indicated by black while negative correlation was indicated by grey lines with the relative significance of each weight in proportion to the line thickness; (**C**) heat map of categorized expression of hub genes in association with all selected patients. The patients number were shown at the horizontal line whereas the categorized feature at the left vertical line (gene expression); the positive feature weight was marked by green and the negative by red.

Increasingly values of AI have been recognized in association with prediction models and data interpretation. Therefore, ANN analysis was introduced to build a model of hub genes using Garson’s algorithm. Specifically, the weights of hub genes in this model are more analogous to the coefficients of linear model, given the fact that large number of adjustable weights could lead to nonlinear effects with challenging interpretation. The relative importance of each node was represented by the combined effects of weight. In fact, the relative importance of each hub gene was determined by dissecting the model weights and reflected by a value ranging from 0 to 1 via the R package, NeuralNetTool. Of note, ALB and TF were the most important predictors of metastasis (Figure 7A). Next, the hub genes were input for the construction of neural network (Figure 7B). In this network, five neurons were identified in hidden layer. The output layer, O1, referred to tumor status (primary or metastasis). Black lines showed positive weighted connections while grey showed negative connections. Bias terms were displayed using an independent neuros, B1 and B2, serving as the intercept of a linear model. Moreover, to visualize the correlation between categorized features and all cases, a facetted heat map was illustrated for the feature weights between primary tumor and metastasis groups. Intriguingly, the expression of ALB≤7.44 showed the highest support to the primary tumor whereas 10.64<ALB≤15.97 and ALB>15.97 both supports the metastasis (Figure 7C). Meanwhile, TF≤7.54 showed the highest negative correlation to the primary tumor (Figure 7C). In addition, FGG≤2.32 supports the primary tumor whereas 6.42<FGG≤11.36 and FGG>11.36 showed supports for metastasis (Figure 7C).

## Discussion

The present study identified potential pathways and genes associated with the tumorigenesis and metastasis in SI-NETs by re-analyzing the gene expression profile of GSE65286. Compared with the original study by Andersson et al. [5], the present study focused on the DEGs in tumor process with multiple bioinformatics strategies, including GSEA and ANN. Intriguingly, Notch signaling (*P*-value<0.001, ES = 0.61) was the top enriched gene sets in T compared with N group. Commonly, Notch1 signaling is absent from NETs and its significant tumor suppressor role has been confirmed [23]. In fact, Notch signaling pathway has been identified as a key regulator for the neuroendocrine differentiation in gastrointestinal carcinoid tumors by inducing the Notch effector hairy and enhancer of split 1 (Hes1) and reducing the achaete–scute complex homolog-like 1 (Asc11/hASH1) [24]. Furthermore, overexpression of Notch signaling also significantly reduced serotonin concentration and corresponding serotonin-reactive cells as well as the expression of tryptophan hydroxylase 1 [24].

Noteworthy, the hub genes from the DEGs between N and T groups (F2, GCG, NTS, CFTR, and APOB) displayed marked correlational alterations (Figure 6A,B), which further highlighted the potential mechanistic insights. In fact, CFTR has been involved in the modulation of neurosecretory activity of the pulmonary neuroendocrine cell and neuroepithelial bodies O_2_ sensor functions [25]. The role of CFTR in SI-NETs remains to be disclosed. Interestingly, our study indicated that the correlations of CFTR with the other four hub genes were markedly increased in T compare to N group (Figure 6A,B). Reasonably presume that the aberration of CFTR could be key in the tumorigenesis of SI-NETs via the simultaneous interaction with F2, GCG, NTS, and APOB.

Meanwhile, the hub genes from the DEGs between T and LM groups (ALB, FGG, AHSG, TF, and GC) also showed marked alterations in the correlations, indicating potential mechanisms involved in the tumor metastasis. Given the close correlations in-between the hub genes (ALB, FGG, AHSG, TF, and GC), the present study further employed non-linear mathematical models to delineate the network with ANN algorithms. In fact, the implications of ANN strategy in genetic mechanism remain sparse due to the challenging interpretations and fundamental difference between conventional explanatory models. Nonetheless, ANN is featured by approximating outcomes using powerful description of complex system with minimal errors [19–21]. In fact, the heat map of categorized features facilitates the ANN-based prediction makings. The ANN network models can be insightful evidences for the ultimate decision process. However, given the complex fitting algorithms in ANN-based prediction models, potential over-fitting was not discussed in the present study.

Limitations of the present study included the quality of normal control samples. In fact, the normal intestinal mucosal samples were retrieved from the resected samples of 10 colorectal cancer patients [5]. Of note, Andersson et al. also proposed that normal intestinal mucosal may not be the optimal control for primary tumors in SINET due to lack of sufficient endocrine cell [5]. Moreover, the lack of experimental mechanistic validation for hub gene correlations and ANN analysis also limited the power of the results.

## Conclusion

This bioinformatics analysis provided insights on potential key pathways and genes networks involved in SI-NETs and established an ANN-based hub gene model for metastatic prediction.

## Abbreviations

ANNs: artificial neural networks
BP: biological processes
CC: cellular components
DAVID: Database for Annotation, Visualization and Integrated Discovery
DEGs: differentially expressed genes
GEO: Gene Expression Omnibus
GO: gene ontologies
GSEA: gene set enrichment analysis
KEGG: Kyoto Encyclopedia of Genes and Genomes
MCODE: Molecular Complex Detection
MF: molecular functions
NET: neuroendocrine tumor
PPI: Protein–protein interaction
SI-NET: small intestinal neuroendocrine tumor
